# Isolation, Evaluation, and Identification of Angiotensin I-Converting Enzyme Inhibitory Peptides from Game Meat

**DOI:** 10.3390/foods9091168

**Published:** 2020-08-25

**Authors:** Shiro Takeda, Sakurako Kaneko, Kazuyuki Sogawa, Abdulatef M Ahhmed, Hirofumi Enomoto, Shinpei Kawarai, Kensuke Taira, Wataru Mizunoya, Masato Minami, Ryoichi Sakata

**Affiliations:** 1 Graduate School of Veterinary Science, Azabu University, Sagamihara 252-5201, Japan; s_kaneko@jlia.jp (S.K.); kawarai@azabu-u.ac.jp (S.K.); taira@azabu-u.ac.jp (K.T.); mizunoya@azabu-u.ac.jp (W.M.); minami@azabu-u.ac.jp (M.M.); sakata@azabu-u.ac.jp (R.S.); 2 School of Veterinary Medicine, Azabu University, Sagamihara 252-5201, Japan; 3 Center for Human and Animal Symbiosis Science, Azabu University, Sagamihara 252-5201, Japan; 4 School of Life and Environmental Science, Azabu University, Sagamihara 252-5201, Japan; sogawa@azabu-u.ac.jp; 5 Chemical and Metallurgical Engineering Faculty, Yildiz Technical University, Istanbul 34220, Turkey; latef.ml@gmail.com; 6 School of Science and Engineering, Teikyo University, Utsunomiya 320-8551, Japan; enomoto@nasu.bio.teikyo-u.ac.jp; 7 Advanced Instrumental Analysis Center, Teikyo University, Utsunomiya 320-8551, Japan; 8 Japan Society for Meat Science and Technology, Ebisu 150-0013, Japan

**Keywords:** venison, angiotensin I-converting enzyme (ACE) inhibitory activity, game meat, bioactive peptide, boar meat, gastrointestinal digestion

## Abstract

Game meat has been underutilized, while it offers the potential to diversify not only the human diet but also increase food production and the nutritional value of meat products. This study aimed to determine the angiotensin I-converting enzyme (ACE) inhibitory activities of the digested game meats (venison and boar meat) compared with those of livestock meats (beef and pork). Through the sodium dodecyl sulfate polyacrylamide gel electrophoresis and size chromatography results, we found that the digested products from each meat had different molecular weights. The ACE inhibitory ratio in all tested samples had gradually increased following by the enzyme treatments. ACE inhibitory ratios and the half maximal inhibitory concentration values indicated that digested venison was the most potent inhibitor of ACE activity, followed by the digested boar meat. The level of anserine in digested venison was higher than that in the other meats, but the carnosine level was lower. Through fractionations and liquid chromatography–tandem mass spectrometry analysis, five ACE inhibitory peptides were identified from the digested venison. Of these peptides, Isoleucine-Lysine- Glutamic Acid-Valine-Threonine-Glutamic Acid-Arginine (IKEVTER) demonstrated the highest ACE inhibitory activity. Therefore, the game meat is food that is believed potentially to offer high bioactivities, particularly antihypertensive forces.

## 1. Introduction

Meat is recognized as having high nutritional value; it is a good source of protein and also contains group B vitamins, minerals, and trace elements. The worldwide demand for meat continues to rise, particularly for pork, beef, and chicken. Game meat, known as meat from wild animals, is thought to have health benefits, including low fat and low cholesterol [1]. Some reports have shown high levels of polyunsaturated fatty acids, including essential fatty acids omega-3 and omega-6, in game meat [2,3]. In particular, the omega-3 acids α-linolenic, eicosapentaenoic acid, and docosahexaenoic acid, which might have beneficial effects on consumer health, are present in game meat [4]. However, game meat is currently underutilized, even though it offers the potential to diversify the human diet and increase food production. Venison, one of the game meats, is rich in proteins and primary essential minerals, with a low subcutaneous and infiltrated fat content [1,5,6]. In recent years, there has been an increasing interest in the farming of deer for meat products [7]. Moreover, the meat of the reindeer, which also belongs to the Cervidae family and has a red-colored raw muscle tissue, is also believed to be with low fat and cholesterol, and with high vitamin B12, minerals, and trace elements [8,9,10,11]. Thus, it is important to experimentally assess the health potential of game meat, including venison, consumed as food.

Great attention has been given to the reduction of risk factors for hypertension or high blood pressure, a common, major cardiovascular disease that affects a wide range of people. This disease primarily occurs due to the lifestyle-related habits and gene sets of an individual, and chemical-based medications have been a general method for its control. However, reports of side-effects of antihypertension medications are so common that many scientists believe that nutritional and therapeutic approaches are better. To that aim, the use of functional foods that are rich in bioactive peptides, also termed nutraceuticals, may be considered as substitutes for many chemical medications. Some low-molecular-weight substances in meat are known to have beneficial bioactivities for human health [12,13,14]. These substances have been found to be released from meat and meat products using in vitro gastrointestinal digestion systems [15,16,17]. Meat-derived peptides have an angiotensin I-converting enzyme (ACE) inhibitory functions [18]. ACE plays an important role in the regulation of blood pressure in humans. Thus, the inhibition of ACE is assumed to improve hypertension [19]. Although the bioactivities of game meat or their by-products were reported for their iron-binding activity, antioxidant activity, and neuroprotective activity [20,21,22,23], to the best of our knowledge, few studies have investigated the ACE inhibitory activities of game meat and then compared these activities in different animal species.

The aims of the present study were to isolate, identify, and evaluate the inhibitory activity of ACE from game meat. The analyses were carried out for pork, wild boar, beef, and venison. The meat products were heated and treated by in vitro gastrointestinal digestion, mimicking the process of digestion in the human body. Their digestibility and their ACE inhibitory activities were then assayed. Active substances were investigated in samples that demonstrated the highest ACE inhibitory activity.

## 2. Materials and Methods

### 2.1. Materials

The edible round muscle meats from wild deer (*Cervus nippon*) were obtained from the Komoro City Government of Nagano Prefecture in Japan. Also, the edible round muscle meats of wild boar (*Sus scrofa leucomystax*) were obtained from the hunting association in Tottori Prefecture in Japan. Both game meat types and their products are available throughout those hunting areas for commercial use and personal consumption. Samples were prepared from three individual wild deer and wild boar. For comparison, three round cuts of beef and pork each were purchased from different local markets in Sagamihara, Kanagawa, Japan. To maintain sample quality, they were stored at −20 °C until use and thawed to 4 °C overnight prior to examination.

### 2.2. Preparation of Gastrointestinal Digestions of the Tested Meat In Vitro

Gastrointestinal digestion was performed according to the method previously reported, with some modifications [16,17]. It was assumed the meats were first cooked, then digested by gastrointestinal enzymes after consumption. In order to isolate meat tissues, fatty tissues were first removed with a knife; then, the red meat was minced and homogenized with water (1:2 *w*/*v*) and heated to 70 °C for 30 min (untreated group). Then, the pH was adjusted to 1.8 with HCl, and pepsin (from porcine stomach mucosa, Nakarai Tesque, Kyoto, Japan) was added at a rate (1:1000) (enzyme:protein) and incubated at 37 °C for 2 h. Afterward, to inactivate the pepsin in the pepsin-treated homogenate (pep group), the pH was adjusted to 6.8 with NaOH and the homogenate was boiled for 10 min. Trypsin and pancreatin (both from porcine pancreas, Nakarai Tesque, Kyoto, Japan) were added to the pep group homogenate at the same concentration as that for pepsin and incubated at 37 °C for 2 h. Subsequently, the homogenate was boiled for 10 min to inactive the added trypsin and pancreatin (pep/try/pan group). The homogenate was centrifuged at 6000× *g* for 10 min at 4 °C. Finally, the supernatant was passed through No. 5 filter paper (Toyo Rosi Kaisha, Tokyo, Japan), collected, and stored at −80 °C until further use.

### 2.3. Analysis of the Products of In Vitro Meat Digestion

To determine products of digestion in the pep and pep/try/pan groups, peptides in the samples were measured by a previously reported method, with slight modifications [20]. Briefly, the *o*-phthaldialdehyde (OPA) (Nakarai Tesque) reagent was prepared by dissolving 40 mg of OPA in 1 mL of methanol; it was then mixed with 25 mL of 100 mM sodium tetraborate buffer, 2.5 mL of 20% sodium dodecyl sulfate, and 100 μL of *β*-mercaptoethanol. The volume was adjusted to 50 mL by adding distilled water. Twenty microliters of homogenate was added to 1.5 mL of OPA reagent and incubated for 2 min at room temperature. The absorbance was read at 340 nm using a UV-1800 spectrophotometer (Shimadzu, Kyoto, Japan), with Gly-Leu (Nakarai Tesque) as the standard. To determine the changes in the molecular weight and structure of proteins in the digested samples, sodium dodecyl sulfate-polyacrylamide gel electrophoresis (SDS-PAGE) was performed on 16% acrylamide gels, which were subsequently stained with Coomassie Brilliant Blue solution (Biorad, CA, USA). In addition, size chromatography was carried out using high-performance liquid chromatography (HPLC) with a JASCO LC-1500 intelligent HPLC system (JASCO, Tokyo, Japan). The sample was injected into a column for size exclusion chromatography (Shodex Asahipak GS-320HQ, Showa Denko, Tokyo, Japan). Elution was performed at 30 °C using 50 mM ammonium acetate adjusted to pH 6.7 at a flow rate of 1.0 mL/min. The eluent was monitored at 220 nm with a UV detector (875UV, JASCO). The following molecular weight (MW) references were subjected to size chromatography analysis: bovine serum albumin (MW: 66,000), cytochrome c (MW: 12,400), aprotinin from bovine lung (MW: 6500), and riboflavin (MW: 376). These reagents were purchased from Fujifilm Wako Pure Chemical (Osaka, Japan).

### 2.4. Assay of ACE Inhibitory Activity of the Products of In Vitro Meat Digestion

The digested samples were centrifuged at 10,000× *g* for 15 min, and the supernatants were subjected to an ACE inhibitory assay. ACE inhibitory activity was measured using a previously reported method [24,25]. ACE, a dipeptidyl carboxypeptidase (EC 3.4.15.1) extracted from rabbit lung, was obtained from Sigma Chemical Co. (St. Louis, MO, USA). Hippuryl-L-histidyl-L-leucine (HHL) (Nacalai Tesque) was used as the synthetic substrate. A 40 µL aliquot of each sample solution was added to 100 µL of borate buffer (containing 0.3 M NaCl, 5 mM HHL, pH 8.3) and incubated at 37 °C for 3 min. Then, 20 µL ACE solution (0.1 U mL^−1^) was added to each sample solution, and the reaction mixture was incubated at 37 °C for 30 min. After that, the reaction was terminated using 250 µL of 1 M HCl, and hippuric acid was extracted by adding 1.5 mL of ethyl acetate and shaking. Eventually, mixtures were centrifuged at 1750× *g* for 10 min, 1 mL of supernatant was transferred into a test tube, and ethyl acetate was eliminated by heat evaporation at 95 °C. The precipitate was re-dissolved in 2 mL of deionized water and measured spectrophotometrically at 228 nm, using a UV-1800 spectrophotometer (Shimadzu, Kyoto, Japan). For the analysis of samples fractionated by reversed-phase chromatography, the synthetic peptides were assayed using the modified method reported by Cheung, et al. [26]. Briefly, 50 µL of sample solution, 100 µL of the ACE 0.01 U/mL, and 20 µL of 25 mM HHL were mixed in a 96-well plate and incubated at 37 °C for 40 min. Subsequently, 100 µL of 1 M NaOH, 10 µL 0.2% OPA, and 15 µL 3.6 M phosphoric acid were added, and the fluorescence of samples was measured using the plate-reader POWERSCAN MX (DS Pharma Biomedical Co., Ltd., Osaka, Japan) at an excitation wavelength of 360 nm and emission wavelength of 460 nm. The inhibitory activity (%) was calculated as follows: inhibitory activity (%) = ((Ac – As)/(Ac – Ab)) × 100. Ac is the intensity of the control, as is the intensity of the sample, and Ab is the intensity of the blank. Data are expressed as the 50% inhibitory concentration (IC_50_) for each sample.

### 2.5. Determination of Imidazole Dipeptides in the Digested Meat Products

The imidazole dipeptides, anserine and carnosine, were measured by HPLC. Digested meat samples were centrifuged at 7000× *g* for 5 min. Then, 0.5 mL of the supernatant from each sample was ultra-filtered at 15,000× *g* for 20 min (Nanosep 10K OMEGA; Pall Corp., New York, NY, USA) to obtain the under-10-kDa fractions. Each fraction was adjusted to 0.5 mL and analyzed with the HPLC Agilent SERIES 1100 system (Agilent Technologies Inc., Santa Clara, CA, USA). For the analysis of anserine and carnosine, the tested solution was injected into a reversed-phase column (InertSustain AQ-C18; GL Sciences Inc., Tokyo, Japan). Elution was performed at 30 °C with 0.2 M ammonium dihydrogenphosphate, 0.1 mM 1-pentanesulfonic acid sodium salt, and 4% acetonitrile solution and adjusted to pH 2.0 with HCl at a flow rate of 0.8 mL min^−1^. Anserine and carnosine were detected by measuring the absorbance at 220 nm. A solution containing 5.0 mM L-anserine nitrate (Fujifilm Wako Pure Chemical) and 5.0 mM carnosine (*β*-Alanyl-L-Histidine, Peptide Institute, Inc., Osaka, Japan) was used as the standard.

### 2.6. Purification and Identification of ACE Inhibitory Peptides in the Digested Meat

First, gel filtration chromatography was carried out, which fractionated the digested products of the meat according to their molecular mass. The Sephadex G-25^®^ superfine gel (GE Healthcare, Uppsala, Sweden) was equilibrated with 0.01 N HCl in a 4.5 × 53 cm column. Then, the separation was performed, using 0.01 N HCl as the eluent at a flow rate of 4.0 mL min^−1^ at room temperature, and fractions were collected every 1.5 min using a Bio-collector AC-5750 (ATTO, Tokyo, Japan). All fractions were lyophilized, dissolved in 1.0 mL distilled water, and stored at −80 °C until the ACE inhibitory activity assay was conducted.

Second, the active fraction from size exclusion chromatography in the ACE inhibitory activity assay was re-fractionated using HPLC with a JASCO LC−1500 intelligent HPLC system. The fractionating conditions followed those of a previously published study, with slight modifications [27]. The sample was injected into a reversed-phase column (InertSustain AQ-C18, 4.6 × 250 mm; GL Sciences, Tokyo, Japan). Elution was performed at 30 °C using a linear gradient mobile phase system, from solvent A (distilled water containing 0.1% *v*/*v* trifluoroacetic acid) to solvent B (acetonitrile containing 0.1% *v*/*v* trifluoroacetic acid), at a flow rate of 0.5 mL/min. The gradient program was carried out in 65 min (solvent B 30% at 30 min, solvent B 50% at 60 min, and solvent B 0% at 65 min). The eluent was monitored at 220 nm with a UV detector (875UV, JASCO) to observe the resultant peaks, and the components of the samples were collected every 2 min using a Bio-collector AC-5750 (ATTO). The fractionated samples were lyophilized, dissolved in 1.0 mL distilled water, and stored at −80 °C until the ACE inhibitory activity assay. The active fraction was freeze-dried and used for the next analysis.

Third, the active fraction from HPLC in the ACE inhibitory activity assay was used to identify the amino acid sequences of the peptides, following the protocol of a previously reported study [28]. The lyophilized samples were rehydrated in 10–30 μL of 25 mM Tris-HCl/20% CH_3_CN containing 25 ng/L trypsin (Trypsin Sequence Grade; Roche Diagnostics GmbH, Mannheim, Germany) for 45 min. Following the removal of the unabsorbed solution, the lyophilized samples were incubated in 10–20 μL buffer of 50 mM Tris-HCl/20% CH_3_CN for 20 h at 37 °C. The solution containing digested protein fragments was transferred to a substitute tube, and the peptide fragments remaining in the gel were extracted in 5% formic acid/50% CH_3_CN for 20 min at room temperature [29]. The peptides were injected into a 0.3 × 5 mm L-trap column (Chemicals Evaluation and Research Institute, Saitama, Japan) and a 0.1 × 50 mm Monolith analytical column (AMR, Tokyo, Japan) attached to an HPLC system (Nanospace SI-2; Shiseido Fine Chemicals, Tokyo, Japan). The flow rate of the mobile phase was 1 μL min^−1^. The solvent composition of the mobile phase was programmed to change in 35 min cycles, with varied mixing ratios of solvent A (2% *v*/*v* CH_3_CN and 0.1% *v*/*v* HCOOH) to solvent B (90% *v*/*v* CH_3_CN and 0.1% *v*/*v* HCOOH). The gradient system of the mobile phase was as follows: 5–50% solvent B for 20 min, 50–95% solvent B for 1 min, 95% solvent B for 3 min, 95–5% solvent B for 1 min, and 5% solvent B for 10 min. Purified peptides from HPLC were introduced into an LTQ-XL ion trap mass spectrometer (Thermo Scientific, San Jose, CA, USA) via an attached Pico Tip (New Objective, Woburn, MA, USA). MS and MS/MS peptide spectra were measured in a data-dependent manner. The MASCOT search engine (Matrix Science, London, UK) was used to identify proteins from the mass and tandem mass spectra of the peptides. Peptide mass data were matched by searching the NCBI database using the MASCOT engine. The minimum significant threshold level for the probability-based MASCOT/MOWSE score was set at 5% [30]. A search of ACE inhibitory peptides was carried out using BIOPEP and the Anti-Hypertensive Inhibiting Peptide Database (AHTPDB) [31].

Peptides were synthesized by Scrum Inc. (Tokyo, Japan). The synthetic peptides were purified using an HPLC column with 98% purity.

### 2.7. Statistical Analyses

All measurements were conducted in triplicate, with each replicate employing a different individual sample. The data are expressed as the mean ± standard deviation (SD, *n* = 3). The results were analyzed by one-way analysis of variance (ANOVA), followed by Tukey’s test. A *p*-value of less than 0.05 was defined as statistically significant.

## 3. Results and Discussion

### 3.1. Products Released from the Digestion of the Tested Meats

**Figure 1:**
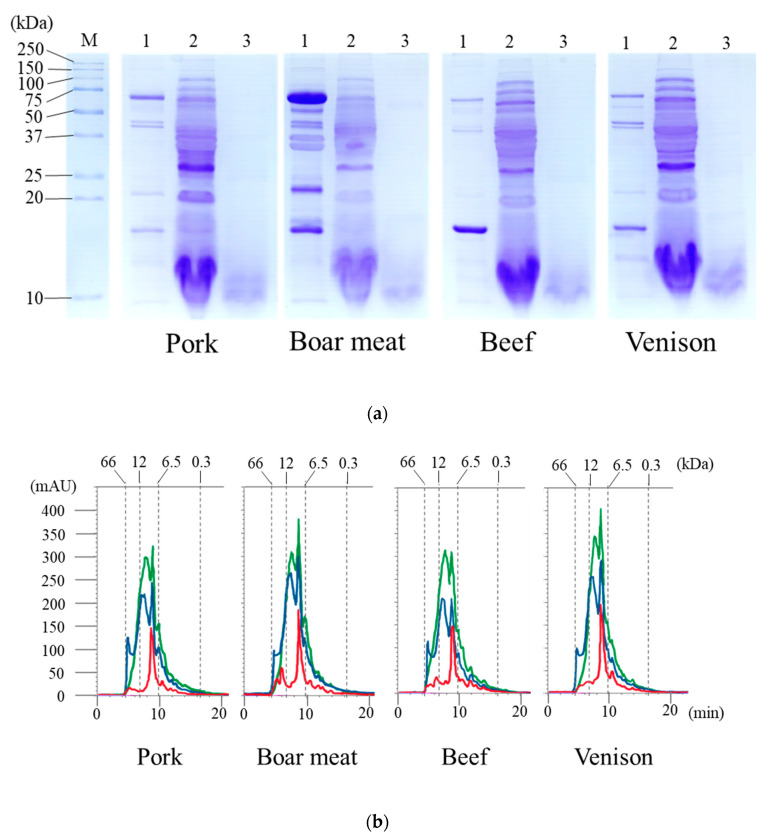
Sodium dodecyl sulfate polyacrylamide gel electrophoresis (SDS-PAGE) profile and chromatograms of the digested meat using size exclusion chromatography. (**a**) SDS-PAGE results of the digested meat. (M) is the lane with the marker. (1), (2), and (3) are the lanes for untreated, pep, and pep/try/pan groups, respectively. (**b**) Chromatograms of digested meats. The lines in red, blue, and green are the chromatograms of untreated, pep, and pep/try/pan groups, respectively.

Several approaches of bioactivity assay have been performed on hydrolysates obtained by enzymatic hydrolysis to determine whether they can detect new peptides in meat products [32]. In the current study, we subjected the tested meats to in vitro gastrointestinal digestion. The peptide concentrations in the untreated, pep, and pep/try/pan groups for all tested meats are shown in Table 1. The untreated group was prepared to yield the water-soluble peptide by cooking. The pep and pep/try/pan groups were obtained for the released peptides, which mainly comprised of aromatic amino acids, and for the released furthermore peptides, respectively. All peptide concentrations in the tested meat samples were significantly elevated in the pep group and further increased in the pep/try/pan group compared to those in the untreated group. For each treatment, there was no difference between the digested meat samples from the different animal species. The water-soluble extracts from cooked pork, boar meat, beef, and venison were subjected to SDS-PAGE (Figure 1a), and their banding patterns for water-soluble proteins (each lane labeled:1) were almost identical. However, boar meat showed a distinctive expression of proteins, with the band at 75 kDa being much larger than that of meat from the other species. In addition, the boar samples displayed a greater number of proteins compared to the others, especially in the range of 36–75 kDa. The pep groups for all tested meats (each lane labeled: 2) were generally similar with respect to their banding patterns, but the intensities of some bands, such as those of the protein bands from 25–37 kDa, were different between groups. It was thought that pepsin in some digested meats might interfere to cleave the proteins at aromatic amino acid residues. Proteins in the pep/try/pan group of each meat type (each lane labeled 3) had completely disappeared and had been digested to form components <20 kDa in weight. To investigate the products of digestion, the water-soluble extracts of the pep and pep/try/pan groups in the pork, boar meat, beef, and venison were subjected to a size chromatography analysis (Figure 1b). The signal intensities from the chromatogram of digested groups in each meat type were elevated from 6.5–12 kDa, compared to those observed within the first 10 min of elution.

It was previously reported that the percentages of meat protein digested by pepsin and trypsin are similar among farmed species, including pork and beef [33]. We also found that the digestibilities of boar meat and venison, as well as those of pork and beef, in pep and pep/try/pan were almost the same; there were no significant differences in peptide concentrations (Table 1). Moreover, the results obtained through SDS-PAGE in the pep group were consistent with those obtained through size chromatography in the pep and pep/try/pan groups (Figure 1a,b). The peptide profiles of meat protein digested by pepsin and trypsin have been reported to be different among meat species due to a difference in amino acid contents [33,34], as well as due to chemical bonds and forces, such as hydrophobic and disulfide interactions. There might be other extrinsic factors leading to differences in the muscle and muscle protein structures between different tested animal species, such as diet and feed, breeding regime, age, and the physical and social stress of pre-slaughter or hunting. Although the patterns of molecular products released by treatment with pep and/or pep/try/pan appeared to be similar between the different meats, the substances presented in a molecular weight might be different in this study. In effect, the expression levels of those substances were consistent, but the number or type of the amino acids would be diverse, which resulted in differing molecular weights of the substances. Hereby, it is believed that the digested meat products in this study had specific peptide sequences and characteristic bioactivities, including ACE inhibitory activity.

### 3.2. ACE Inhibitory Activity and Imidazole Dipeptide Content in the Digested Meat Products

The ACE inhibition ratios induced by the water-soluble extract and its digestion products are shown in Table 2. The percentage of ACE inhibition in each meat sample was significantly elevated in the pep and pep/try/pan groups compared to that in the untreated group (*p* < 0.05). The highest percentage inhibition for pork and venison was observed in their pep/try/pan groups. Inhibition rates for venison in the pep/try/pan groups were significantly higher than those in the pep group (*p* < 0.05) and significantly higher than those for the other meat samples (*p* < 0.05). Boar meat and beef showed the highest inhibition ratios in their pep groups and the lower inhibition ratios in their pep/try/pen groups, but the difference was not significant. The percentage inhibition of ACE by boar meat in the pep group was significantly higher than that of pork and beef (*p* < 0.05). The pep/try/pan-treated samples were assayed to determine their IC_50_ value, the concentration of the generated peptides required to reach 50% inhibition of ACE. The IC_50_ values of pork, beef, wild boar meat, and venison in the pep/try/pan group were 0.48 ± 0.07, 0.58 ± 0.12, 0.41 ± 0.10, and 0.30 ± 0.09 mM, respectively. Notably, the IC_50_ value for venison treated by pep/try/pan was significantly lower than that of the other digested meats (*p* < 0.05).

To investigate whether the imidazole dipeptides (anserine and carnosine) of the digested meat products affected the ACE inhibitory activities in this study, their levels were measured in each pep/try/pan group. The concentrations of imidazole dipeptides varied quite remarkably between meat species (Table 3). The anserine levels of the digested beef, boar meat, and venison were significantly higher than those of digested pork (*p* < 0.05). In particular, the anserine level of digested venison was significantly higher than that of all the other tested meats (*p* < 0.05). The concentration of anserine in venison was 10-fold higher than that in pork. However, the carnosine levels in the digested pork and beef were significantly higher than those in the digested boar meat and venison (*p* < 0.05), and the level in venison was the lowest among the levels in the tested meats; this result was opposite to that for anserine.

Previous studies have shown that proteolysis by enzyme digestion could trigger the generation of many bioactive peptides, including ACE inhibitory peptides with low molecular weights [18]. In this study as well, we observed a significant elevation of ACE inhibitory activity in the pep and pep/try/pan groups for all tested meats compared to those of the untreated groups (*p* < 0.05). Jensen et al. reported that the ACE inhibitory activities did not significantly differ between pork and beef, which is consistent with the results in this study [35]. However, several other studies have shown different tendencies: the difference of ACE inhibitory activities between the digested pork and beef was observed. One of the reasons might be that the in vitro digestion was conducted in several different procedures and conditions. The standardized in vitro food digestion protocol was recently published [36]. Thus, it would be expected to be used to prepare the digested meat products in further studies. Meanwhile, boar meat and venison showed higher ACE inhibitory percentages after pep/try/pan digestion than pork and beef, and the IC_50_ value of venison in the pep/try/pan group was significantly lower than those in the pep/try/pan groups of other digested meats. Livestock meat and game meat differ in physicochemical quality [1,5]. In addition, the chemical composition of raw meat also differs between animal species [5,37]. Thus, the differences in physicochemical quality between livestock meat and game meat might cause differences in the compositions of digested products and their ACE inhibitory activities. In addition, the ACE inhibitory activities of the game meats including boar meat and venison are potentially higher than those of pork and beef, which might be the cause of their greater in vivo antihypertensive activity.

Imidazole dipeptides, including anserine and carnosine, are known to be bioactive components of meat and demonstrate ACE inhibitory activity [12,38,39]. In addition, it was reported that the anserine and carnosine levels were different among meats from various farm animal species; the carnosine levels of pork and beef were clearly higher than those of chicken, sheep, and turkey, but the anserine levels of pork and beef were lower [40,41]. In this study, the levels of carnosine in digested pep/try/pan groups of all tested meats were higher than those of anserine. Although, the carnosine level of venison treated by pep/try/pan was the lowest in the tested meats, the anserine level of venison treated with pep/try/pan was the highest among the tested meats; this result is consistent with the results of ACE inhibitory activity. Thus, anserine is potentially an active substance, however, the other peptide might have contributed to express the high ACE activity in the venison treated with pep/try/pan.

### 3.3. Purification and Identification of ACE Inhibitory Peptides in the Digested Venison

**Figure 2:**
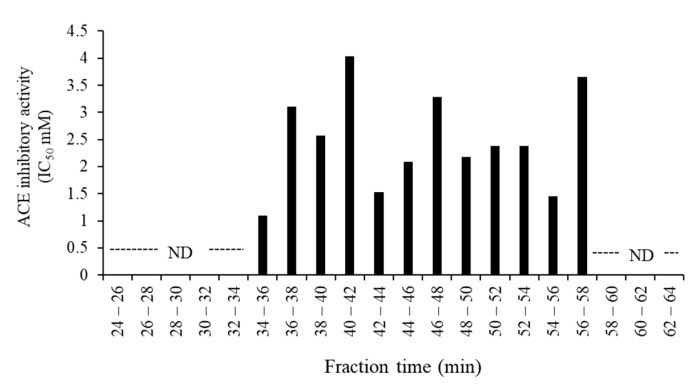
Angiotensin I-converting enzyme (ACE) inhibitory activity of the fractions of digested venison. The venison digested with pepsin, trypsin, and pancreatin was fractionated using gel filtration chromatography. The ACE inhibitory activity in each fraction was assayed. The data were expressed as mean in triplicates. ND in the figure indicates ‘’not detectable’’.

**Figure 3:**
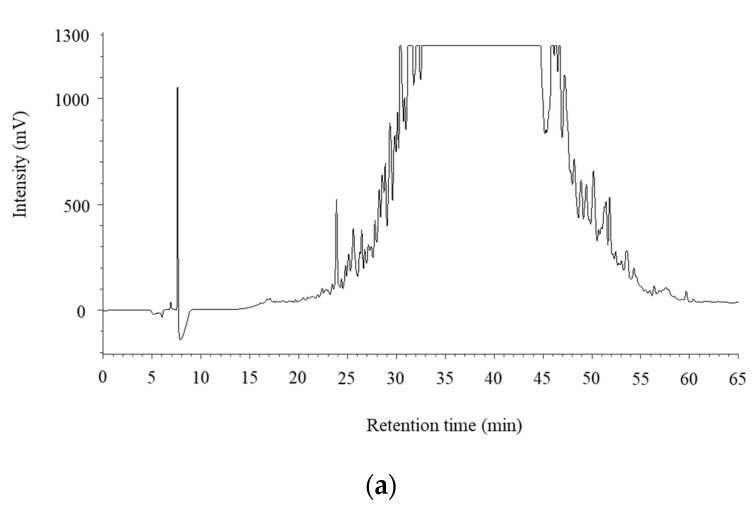

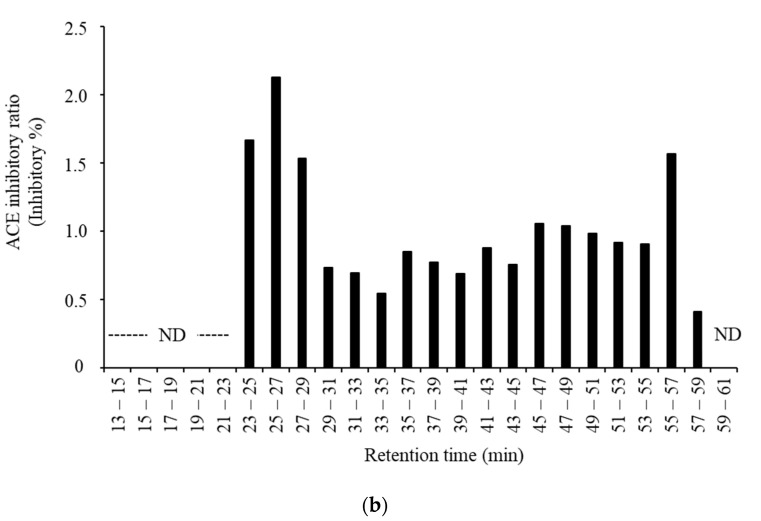
Chromatogram in reversed-phase HPLC and the angiotensin I-converting enzyme (ACE) inhibitory activity of fraction of the digested venison. (**a**) Chromatogram in reversed-phase HPLC using the fraction at 34–36 min by gel filtration chromatography. (**b**) ACE inhibitory activity of the fractions using reversed-phase HPLC. The data are expressed as the means of triplicates. ND in the figure indicates ‘’not detectable’’.

Bioactive peptides, including those with antihypertensive activity, have sequences of 2–30 amino acids [42,43]. They have been identified in a wide variety of foods, including milk; muscle sources, such as beef, chicken, pork; marine sources, but not so extensively in game meat. To investigate the peptides derived from digested venison in the pep/try/pan group, which showed the highest ACE inhibitory activity, it was subjected to gel filtration chromatography using a Sephadex G-25 column. The ACE inhibitory activity of each fraction is shown in Figure 2. The results show that the fraction eluted at 34–36 min after the loading of the sample had the greatest ACE inhibitory activity; its IC_50_ value was the lowest among all tested fractions. This eluted fraction was subjected to reversed-phase chromatography in an HPLC system. The chromatogram obtained for the samples fractionated by HPLC is shown in Figure 3a, then the 24 fractions were assessed for ACE inhibitory activity. As shown in Figure 3b, the fraction eluted at 25–27 min after loading had the greatest ACE inhibitory ratio. The next highest ACE inhibition ratios, in descending order, were observed in fractions obtained at 23–25, 55–57, and 27–29 min.

To identify the source of the ACE inhibitory activities, the most active fraction (eluted at 25–27 min) was subjected to LC-MS/MS analysis. According to the results and using a database, five peptides were selected for analysis for amino acid identification and to determine from which proteins they originated (Table 4). In addition, the identified peptides were synthesized, and their ACE inhibitory activities were assayed (Table 4). All the synthesized peptides showed the ACE inhibitory activities, except for the peptide SEIQAALEEAEASLEHEEGK which was derived from myosin-1. IKEVTER is another myosin-1 peptide, which demonstrated great activity for ACE inhibition and could be considered a valuable ACE inhibitory peptide, with an IC_50_ value of 86.52 μg/mL, which was significantly lower than those of the other synthesized peptides. Moreover, this peptide sequence has not yet been observed in the bioactive peptide databases at BIOPEP and AHTPDB.

The potent ACE inhibitory peptides are generally short chains of 3–6 amino acids, with a Ser, Leu, or Thr at the C-terminal, which plays an important role in this activity [31]. The ACE inhibitory peptides identified in this study, except for IKEVTER, seem to be of a larger size than those of inhibitory peptides described in previous reports. ACE inhibitory peptides have been previously reported from meat and seafood vertebrate specimens, and their IC_50_ values have been reported [18]. The ACE inhibitory peptides derived from meat do not always include a Ser, Leu, or Thr at the C-terminal. The peptide ‘IKEVTER’ which had high ACE activity in the present study, also does not consist of those amino acids. Many peptides, with various amino acid sequences, have shown strong ACE inhibitory activity, including ND, TK, GFHI, DFHING, FHG, and GLSDGEWQ, with ACE inhibition IC_50_ values of 3.9, 112, 117, 64.3, 52.9, and 50.5 μg/mL, respectively [18,44,45]. Since the IC_50_ value of the peptide IKEVTER was 86.52 μg/mL, its activity was thought to be in an acceptable range for ACE inhibition, compared to those of other reported peptides. Therefore, the IKEVTER peptide is considered a novel ACE inhibitory peptide from the venison treated with pep/try/pan.

## 4. Conclusions

The ACE inhibitory activities were different among the samples digested with pep and/or pep/try/pan in each tested meat species. In particular, venison in the pep/try/pan group demonstrated the highest ACE inhibition ratio and the lowest IC_50_ value. The digestibilities of meat tested by pep and pep/try/pan were similar, and there were no significant differences in the peptide levels of different digestion groups. Thus, the components of digested products might be distinctive for each tested meat species. Although the carnosine level in venison in the pep/try/pan group was lowest among the tested meats, the anserine level was the highest. Moreover, the peptide IKEVTER was identified as a novel ACE inhibitory active substance in the venison treated by pep/try/pan. Based on these results, the present study provides an insight into the release of antihypertensive peptides in game meat, using the peptic digestion process. In particular, our findings suggest that venison has ACE inhibitory activities. Therefore, venison can be described as a meat with high bioactivity as well as with low fat, low cholesterol, and high polyunsaturated fatty acids. Further studies are necessary, using a live animal model, on the bioavailability of the novel peptide identified from pep/try/pan-digested venison, and to prove its efficacy for lowering blood pressure. In addition, the ACE inhibitory activity of farmed venison should be investigated further.

## Figures and Tables

**Table 1:** Peptide concentrations in the products of digested meats.

	Peptide Concentration (mM)		
	Untreated	Pep	Pep/Try/Pan
Pork	22.28 ± 0.99 ^a^	52.80 ± 6.65 ^b^	80.10 ± 5.52 ^c^
Boar meat	20.32 ± 1.36 ^a^	58.03 ± 6.50 ^b^	78.45 ± 8.09 ^c^
Beef	23.56 ± 1.83 ^a^	51.62 ± 1.89 ^b^	78.95 ± 3.28 ^c^
Venison	22.20 ± 2.00 ^a^	50.56 ± 2.37 ^b^	75.70 ± 4.87 ^c^

The mean and standard deviation of three independent experiments are presented. Values with different superscripts indicate a significant difference in each digested group, as determined by one-way ANOVA, followed by Tukey’s test (*p* < 0.05).

**Table 2:** Angiotensin I-converting enzyme (ACE) inhibitory activities in the digested meat products.

Meat Type	ACE Inhibitory Percentage (%)		
	Untreated	Pep	Pep/Try/Pan
Pork	43.71 ± 5.42 ^a, A^	64.36 ± 1.00 ^b, A^	65.00 ± 6.43 ^b, A^
Boar meat	44.67 ± 5.03 ^a, A^	79.44 ± 1.70 ^b, B^	78.04 ± 2.47 ^b, B^
Beef	47.18 ± 5.90 ^a, A^	64.15 ± 2.81 ^b, A^	63.20 ± 3.26 ^b, A^
Venison	52.60 ± 2.89 ^a, A^	77.29 ± 5.96 ^b, AB^	89.38 ± 5.57 ^c, C^

The sample was prepared at a 10 mM peptide concentration for assaying ACE inhibitory activity. The mean and standard deviation of three independent experiments are presented. Different small letters indicate a significant difference in each tested meat group, as determined by one-way ANOVA, followed by Tukey’s test (*p* < 0.05). Different large letters indicate a significant difference in each enzyme-digested group, as determined by one-way ANOVA, followed by Tukey’s test (*p* < 0.05).

**Table 3:** Imidazole dipeptide content in the digested meat products.

Meat Type	Anserine (μM)	Carnosine (μM)
Pork	66.67 ± 1.15 ^a^	2750 ± 100 ^a^
Beef	323.33 ± 7.02 ^b^	2190 ± 40 ^a^
Boar meat	401.33 ± 9.45 ^c^	1730 ± 30 ^b^
Venison	668.67 ± 1.15 ^d^	1040 ± 10 ^c^

The mean and standard deviation of three independent experiments are presented. Values with different superscripts indicate a significant difference in a column of anserine or carnosine by one-way ANOVA, followed by Tukey’s test (*p* < 0.05).

**Table 4:** Identified and synthesized peptides from the digested venison and their angiotensin I-converting enzyme (ACE) inhibitory activities.

Peptide Sequence	Origin	Ace Inhibitory Activity (IC_50_: μg/mL)
VcNYVNWIQQTIAAN	Tropomyosin alpha-3 chain	358.64 ± 64.71 ^a^
mQGTLEDQIISANPLLEAFGNAK	Myosin-1	292.83 ± 38.27 ^a,b^
IKEVTER	Myosin-1	86.52 ± 18.84 ^c^
TEAGATVTVK	Myosin-1	208.64 ± 5.57 ^b^
SEIQAALEEAEASLEHEEGK	Myosin-1	ND

The means and standard deviations of three repeated experiments are presented as the results of ACE inhibitory activity. Values with different superscripts indicate a significant difference compared to other values in the column, as determined by one-way ANOVA, followed by Tukey’s test (*p* < 0.05). ND indicates “not detectable”.
